# Distribution of endemic angiosperm species in Brazil on a municipality level

**DOI:** 10.3897/BDJ.9.e66043

**Published:** 2021-06-03

**Authors:** Janaína Gomes-da-Silva, Joâo Lanna, Rafaela Campostrini Forzza

**Affiliations:** 1 Instituto de Pesquisas do Jardim Botânico do Rio de Janeiro, Rio de Janeiro, Brazil Instituto de Pesquisas do Jardim Botânico do Rio de Janeiro Rio de Janeiro Brazil

**Keywords:** Endemic species, data re-use, flowering plants, occurrence records, primary biodiversity data, South America

## Abstract

**Background:**

Herbarium collections and the data they hold are the main sources of plant biodiversity information. These collections contain taxonomical and spatial data on living and extinct species; consequently, they are the fundamental basis for temporal and spatial biogeographical studies of plants. Mega projects focused on providing digital and free access to accurate biodiversity data have transformed plant science research, mainly in the past two decades. In this sense, researchers today are overwhelmed by the many different datasets in online repositories. There are also several challenges involved in using these data for biogeographical analyses. Analyses performed on the data available in the repositories show that 70-75% of the total amount of data have spatial deficiencies and a high number of records lack coordinates. This shortage of reliable primary biogeographical information creates serious impediments for biogeographical analyses and conservation assessments and taxonomic revisions consequently produces obstacles for evaluations of threats to biodiversity at global, regional and local levels. With the aim of contributing to botanical and biogeographical research, this paper provides georeferenced spatial data for angiosperm species endemic to Brazil. The information from two reliable online databases, i.e. the Flora do Brasil 2020 floristic database (BFG) and Plantas do Brasil: Resgate Histórico e Herbário Virtual para o Conhecimento e Conservação da Flora Brasileira (REFLORA), which are both based on records collected over the course of the last two centuries, is used to create this spatial dataset.

**New information:**

We provide three taxonomically-edited and georeferenced datasets for basal angiosperms, monocots and eudicots, covering a total of 14,992 endemic species from Brazil. Producing this consolidated dataset involved several months of detailed revision of coordinates and nomenclaturally updating of the names in these datasets. The information provided in this geo-referenced dataset, covering two centuries of specimen collections, will contribute to several botanical and mainly biogeographical studies.

## Introduction

Herbarium collections and the data they hold have been one of the main sources of plant biodiversity information through time (Gasper et al. 2020). They include taxonomical and spatial data on living and extinct species and, therefore, provide a fundamental basis for both temporal and spatial studies of plants (Funk 2003, Hortal et al. 2015, James et al. 2018). Mega projects, focusing on providing accurate, digital open-access data on biodiversity, including digitised specimens and species occurrence data, have transformed biodiversity analysis in the past two decades (Graham et al. 2004, La Salle et al. 2016). In this sense, today’s large amount of different datasets in online repositories can be overwhelming, for example, the Global Biodiversity Information Facility (https://www.gbif.org/pt/,GBFI 2021), the Flora of Brasil 2020 floristic database (BFG 2020) and the Plantas do Brasil: Resgate Histórico e Herbário Virtual para o Conhecimento e Conservação da Flora Brasileira (http://reflora.jbrj.gov.br/, REFLORA 2021) virtual herbarium.

Widespread access to taxonomic and distributional data is producing great advances in botanical and biogeographical research, as well as supporting more accurate evaluations of extinction risks (Gomes‐da‐Silva and Forzza 2020, Robiansyah and Wardani 2020). In spite of this substantial progress, there are several challenges and limitations when applying open-access repository data (see discussion in James et al. 2018). Unfortunately, the quality of the species occurrence records available in most collections is low (Robertson et al. 2016). Evaluations of the data available in repositories show that ca. 70-75% of these data have spatial deficiencies, mainly with regard to the georeferencing quality (Colli‐Silva et al. 2020, Jin and Yang 2020, Marcer et al. 2020). Jin and Yang (2020) assessed 30,242,556 occurrence records from different repositories and demonstrated that only 28% of the records had high-quality taxonomic and spatial data. In addition, analyses have shown that erroneous records, containing geographic inaccuracies, affect the spatial patterns for species more significantly than taxonomic uncertainties (Maldonado et al. 2015). The spatial accuracy of the data available in the GBIF database for flowering plants in the Brazilian Atlantic Forest was evaluated recently and the analysis revealed that only 25% of the records contained precise spatial information (Colli‐Silva et al. 2020). Similarly, an analysis of the REFLORA 2021 database for the present work showed that the georeferenced data have repetitive errors, of which the most common are missing coordinates (lat/long), zero values entered for the latitude and longitude, points in the oceans (for terrestrial species) or the Antarctic, only the latitude or longitude coordinates entered and lack of coordinate precision. These analyses (Colli‐Silva et al. 2020, Jin and Yang 2020, present work) reveal the value in cleaning data in biodiversity studies and the need to georeference these databases.

Manipulating millions of records is an extremely complicated task. In recent years, workflows, tools and methods have been developed for dealing with taxonomic and geographic errors, simplifying the process (Chamberlain 2016, Robertson et al. 2016, Zizka et al. 2019, Jin and Yang 2020) by identifying potential geographical and temporal errors in databases and converting the coordinates to various text formats (e.g. Chamberlain 2016, Robertson et al. 2016, Zizka et al. 2019, Jin and Yang 2020). In addition, BDcleaner can be used to remove taxonomic errors (Jin and Yang 2020). However, there is no effective tool for correcting geographical errors in lieu of discarding them.

As manual data cleaning is laborious (Marcer et al. 2020), many studies choose to reduce datasets by discarding occurrence data with correctable geographic errors. This incomplete data sampling introduces uncertainties to analyses and compromises the results, particularly in terms of regional analyses (Hortal et al. 2015; Casagranda and Goloboff 2019). To employ the IUCN Categories and Criteria used to create the (IUCN) Red List for species at risk of extinction, mainly criteria B [severely fragmented] and D2 [very restricted area of occupancy], it is necessary to identify the geographical ranges of species accurately and reliably (IUCN 2012). Up until the last decade, ca. 1% (61,914; IUCN 2012) of species have been evaluated using the Red List to define their conservation status (Bachman et al. 2011). Although the number of species assessed has doubled in the last 10 years (120, 372; IUCN 2020), this number is still far from the IUCN target of 160,000 for 2020 (IUCN 2020).

Brazil has the highest biodiversity of vascular plants on the planet (BFG: Filardi et al. 2018). According to the updated version of the BFG database, there are currently 32,696 species of angiosperms on record in Brazil, of which ca. 18,000 species are endemic to the country (BFG 2020). Despite the errors in spatial data, the high number of records lacking coordinates and the gaps in its database, which are common in all databases (Beck et al. 2013, Jin and Yang 2020), the REFLORA repository, used in conjunction with the Flora of Brasil (BFG 2020), provides reliable data. These two repositories represent massive collaborations of taxonomists from various institutions, including experts on every flora family in the country. Using the filters for the BFG database, it is possible to generate a verified taxonomical list of endemic Brazilian species, carefully prepared by several taxonomists. This task, which seems simple nowadays, was exceedingly difficult or impossible prior to the creation of the BFG database.

The geographical range of a species forms the basis for biogeographical studies. Repositories, such as REFLORA 2021, make distributional records accessible, mitigating the poor geographic data. With millions of high-resolution images, the REFLORA project minimises the deficiencies of primary data (Canteiro et al. 2019). However, most of the data provided by the repository lack georeferencing. Thus, with the aim of contributing to botanical and mainly with biogeographical research in Angiospermae, this paper provides georeferences for 14,992 endemic Brazilian species from 173 families, based on reliable taxonomic data from the REFLORA and BFG datasets.

## Project description

### Title

Geo-referenced spatial data for angiosperm species endemic to Brazil

### Design description

The REFLORA 2021 and BFG (BFG 2020) databases are fed new data daily and edited for changes in nomenclature. The georeferencing work carried out here was developed between August 2018 and December 2019. Thus, the difference between the number of endemic species recorded in 2020 (i.e. about 18,000 species) and the number of georeferenced species provided here (i.e. 14,992 species) is supported by the following factors:

1. In August 2018, 1,393 species had no vouchers in REFLORA.
2. In order to obtain the highest possible accuracy in species occurrence data, we established editing procedures for the use of geographical distributions from the collection records (outlined below). These procedures made it impossible to include 1,615 species with inconsistencies in the collection records.

This georeferenced occurrence dataset for endemic species provides the basis for a wide range of biodiversity studies, for example, spatial studies conducted at various hierarchical levels, i.e. family, genus, species; effects of global change; changes in distributions of species; conservation; and systematics.

### Funding

Conselho Nacional de Desenvolvimento Cientıfico e Tecnológico (CNPq) and FAPERJ - Fundação de Amparo à Pesquisa do Estado do Rio de Janeiro for the postdoctoral fellowship granted to JGS. RCF received a Research Productivity Fellowship from CNPq (proc.303420/2016-2) and FAPERJ (processes n° E-26/202.778/2018) through Programa Cientista do Nosso Estado.

## Sampling methods

### Study extent

**Brazilian angiosperms dataset**

**Figure 1.**
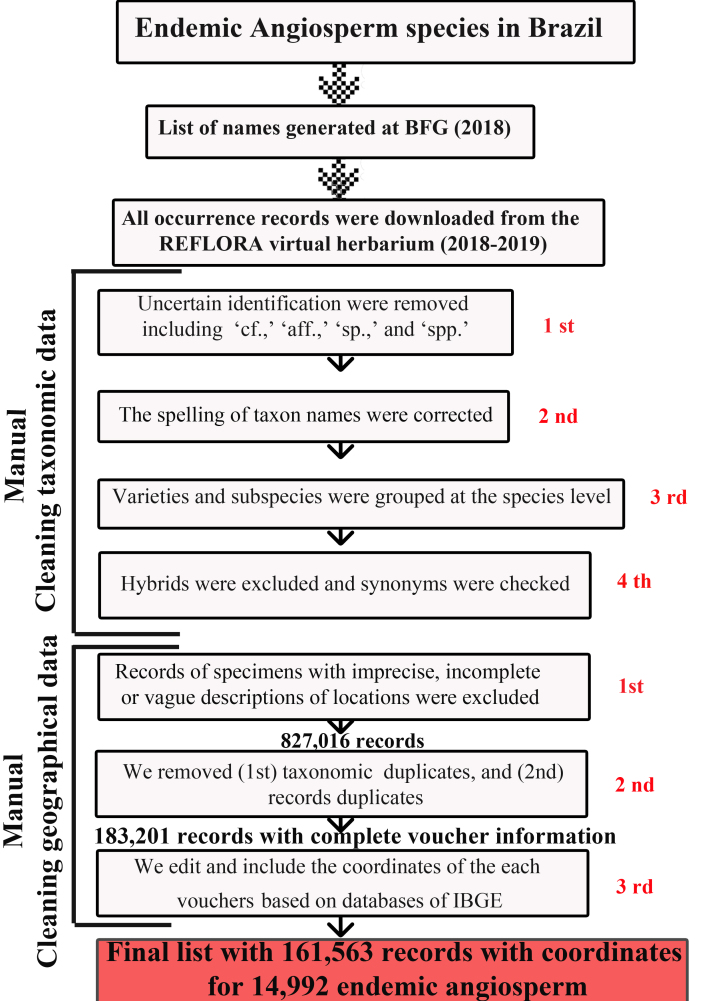
Taxonomic and geographic data refinement workflow. Steps of data filtering to obtain the endemic angiosperm species list for Brazil, based on the list available from BFG 2018 (Filardi et al. 2018), in the Brazilian Flora 2020 website and records from Reflora Herbarium Virtual (2018-2019).

**Species list compilation**:

The list of species was established in two phases. First, the initial list of names of all endemic species of Angiospermae was generated through the BFG in the Brazilian Flora (BFG 2020) website (http://floradobrasil.jbrj.gov.br), edited by several taxonomic experts in each sampled family, using the following search filters: (1) Group: Angiospermae; (2) Occurs in Brazil: yes; (3) Occurrence: only occurs in Brazil; (4) Endemism: only endemic to Brazil; and (5) Origin: native (Fig. 1).

Based on this list of all endemic Brazilian angiosperm species retrieved from the BFG floristic database between August 2018 and October 2019, all occurrence records were downloaded from the REFLORA 2021 virtual herbarium (www.reflora.jbrj.gov.br) from 73 herbaria (ALCB, ASE, B, BRBA, CEN, CEPEC, CESJ, CGMS, COR, CRI, DVPR, E, EAC, ECT, ESA, EVB, FIG, FLOR, FURB, GH, HACAM, HBR, HCF, HDCF, HEPH, HRCB, HSTM, HTO, HUCO, HUCP, HUEFS, HUEM, HUEMG, HUENF, HUFU, HUNEB, HUNI, HUPG, HVASF, IAN, IBGE, ICN, K, LUSC, MAC, MBM, MBML, MG, MO, MUFAL, NY, P, PEL, PMSP, R, RB, RBR, REAL, RFA, UERJ, RFFP, RON, S, SJRP, SPF, UB, UFRN, UNIP, UNOP, UPCB, US, VIES and W, the herbaria acronyms following Thiers (2020), continuously updated). After these two phases, 18,000 endemic species to Brazil were identified, corresponding to the raw database. Producing this consolidated dataset involved 1 1/2 years of detailed revision of coordinates and nomenclaturally updating of the names in these datasets, as follows:

We created a protocol to clean the datasets (Fig. 1), the data were processed carefully by checking nomenclatural status and excluding records with erroneous occurrence data. The accuracy of species identification follows the list of endemic species of BFG 2020. Four steps were conducted for cleaning the taxonomic data. In the first step, we checked and cleaned the data taxonomically and nomenclaturally; only vouchers identified to species level about which we were uncertain were removed, including ‘cf.,’ ‘aff.,’ ‘sp.,’ and ‘spp.’. In the second step, we corrected the spelling of taxon names, which, for some species, had multiple entries with different spellings. In the third step, varieties and subspecies were grouped at the species level. In the fourth step, hybrids were excluded, synonyms were checked and accepted names were adopted according to the BGF. We performed the first four steps using the “filter” tool in Microsoft Excel v. 14.5 (Microsoft Office 2010 Proofing Tools).

Subsequently, we conducted manual cleaning procedures on the records. For cleaning the records, three steps were performed on the geographic data. In the first step, records of specimens with imprecise or vague descriptions of locations (e.g. Negro River, north coast, south coast) and incomplete (e.g. Amazonia, Bahia, Brazil) or incongruent information concerning locations (e.g. with no administrative unit, location in the ocean) were excluded. In the second step, we removed the taxonomic duplicates and records of duplicate samples with the same species name and place of occurrence and voucher information. In the final dataset, each record corresponds to a single herbarium specimen for which the geographical location has been checked and is unique to that locality. Duplicates were removed from the list, based on locality, collector name, collector number and the year in which the sample was collected. After data cleaning, the total number of records dropped from 827,016 to 183,201 occurrence records with complete voucher information.

The use of GPS became more widespread in 1995-1996, but there were still few satellites at that time (Kaplan 2005). Given that the occurrence records for all species endemic to Brazil were collected mainly over the last two centuries, it was not surprising that more than 75% records were not georeferenced. Hence, in the third step, we manually edited and included the coordinates of each voucher, based on databases of localities and municipalities maintained by the Brazilian Institute of Geography and Statistics website (IBGE) (http://mapas.ibge.gov.br), for 161,563 occurrence records of 14,992 endemic angiosperm species. For 21,632 records, it was not possible to perform georeferencing due to lack of sufficient information on the voucher. In this step, we removed the complete voucher data, since the main objective concerns the use of the dataset for biogeographical analysis. We performed the three steps using the “filter” tool in Microsoft Excel v. 14.5 (Microsoft Office 2010 Proofing Tools).

The final checklist is composed of native and endemic angiosperms and includes only vouchers identified to the species level, based on the Brazilian Flora (BFG 2020) and complete records, based on REFLORA. The complete list of vouchers, including all geographical duplicates (duplicate samples for same location) and photos to check the identity of the species, is available at REFLORA 2021(http://reflora.jbrj.gov.br/).

## Geographic coverage

### Description

**Figure 2.**
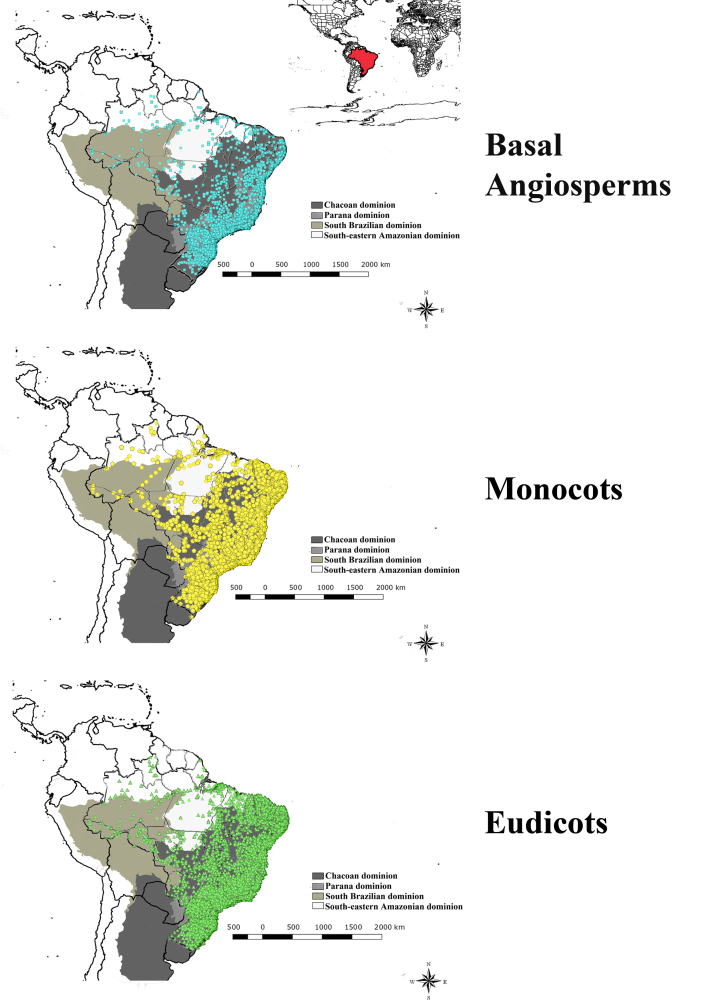
Spatial distribution of angiosperms for all georeferenced data available at the Reflora Herbarium Virtual after data cleaning.

The geographic coverage encompasses the national territory of Brazil, which extends from 5° to -34° Latitude; -34° to -73° Longitude and covers a total area of approximately 8.5 million km² (IBGE). The dataset comprised all species of Angiospermae found exclusively in Brazil and it contains occurrence records in six phytogeographic domains, i.e. Amazonia, Caatinga, Cerrado, the Atlantic Forest, Pampa and Pantanal, in Chacoan, Parana, South Brazilian and South-eastern Amazonian dominions (Fig. 2, sensu Morrone (2014)).

### Coordinates

-34 and -5° Latitude; -73° and -34 Longitude.

## Taxonomic coverage

### Description

**Figure 3.**
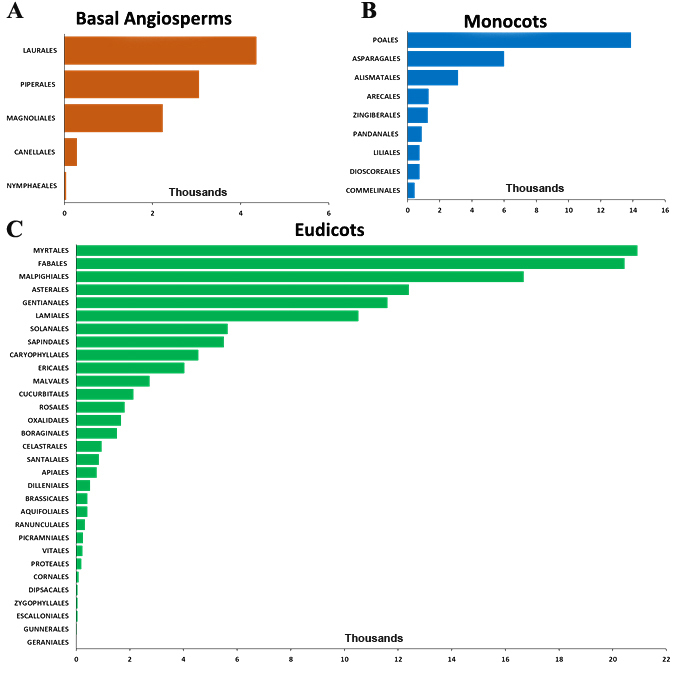
Number of records georeferenced for endemic angiosperm species in Brazil on a municipality level, by order for: **A.** basal angiosperms; **B.** monocots; and **C.** eudicots.

To facilitate the search for taxa at different hierarchical levels, the dataset comprises three different worksheets of specimens collected over the past two centuries organised according to APG IV classification (Chase et al. 2016) and these have been organised alphabetically, as follows:

(1st Worksheet) A total of 649 species of basal angiosperms belonging to five orders, i.e. Canellales, Laurales, Magnoliales, Nymphaeales and Piperales from 13 families and 50 genera. Number of records is georeferenced by order in Fig. 3A.

(2nd Worksheet) A total of 3,854 species of monocots belonging to nine orders, i.e. Alismatales, Arecales, Asparagales, Commelinales, Dioscoreales, Liliales, Pandanales, Poales and Zingiberales from 32 families and 370 genera. Number of records is georeferenced by order in Fig. 3B.

(3rd Worksheet) A total of 10,489 eudicots, belonging to 31 orders, i.e. Apiales, Aquifoliales, Asterales, Boraginales, Brassicales, Caryophyllales, Celastrales, Cornales, Cucurbitales, Dilleniales, Dipsacales, Ericales, Escalloniales, Fabales, Gentianales, Geraniales, Gunnerales, Lamiales, Malpighiales, Malvales, Myrtales, Oxalidales, Picramniales, Proteales, Ranunculales, Rosales, Santalales, Sapindales, Solanales, Vitales and Zygophyllales from 128 families and 1,199 genera. Number of records is georeferenced by order in Fig. 3C.

## Usage licence

### Usage licence

Creative Commons Public Domain Waiver (CC-Zero)

## Data resources

### Data package title

Distribution of endemic angiosperm species in Brazil on a municipality level.

### Resource link

https://ckan.jbrj.gov.br/dataset/mitigating-the-question-of-the-geographic-distribution

### Number of data sets

3

### Data set 1.

#### Data set name

Basal_Angiosperms_Brazil_Gomes_da_Silva_Forzza_Lanna.tsv

#### Data format

TSV

#### Number of columns

8

#### Download URL

https://ckan.jbrj.gov.br/dataset/e1eb798c-601a-4d20-bf17-87dc037ed73e/resource/5ceeb350-b071-46bb-86b4-071b1bbf1372/download/basal_angiosperms_brazil_gomes_da_silva_forzza_lanna.tsv

#### Description

Data containing the geographic distribution of 649 species of basal angiosperms from 13 families.

**Data set 1.** 

Column label	Column description
family	The scientific name of the family in which the taxon is classified.
genus	The scientific name of the genus in which the taxon is classified.
specificEpithet	Scientific name.
country	The country where the species occur.
stateProvince	State of Brazil where species occur.
municipality	Municipality of Brazil where species occur.
decimalLatitude	The latitude component (N/S) of the coordinates of the municipality where the species occur, in decimal degrees.
decimalLongitude	The longitude component (E/W) of the coordinates of the municipality where the species occur, in decimal degrees.

### Data set 2.

#### Data set name

Eudicots_Brazil_Gomes_da_Silva_Forzza_Lanna.tsv

#### Data format

TSV

#### Number of columns

8

#### Download URL

https://ckan.jbrj.gov.br/dataset/e1eb798c-601a-4d20-bf17-87dc037ed73e/resource/d2160257-a141-4ff4-89f2-d93edef0e6a6/download/eudicots_brazil_gomes_da_silva_forzza_lanna.tsv

#### Description

Data containing the geographic distribution of 10,489 eudicots from 128 families.

**Data set 2.** 

Column label	Column description
family	The scientific name of the family in which the taxon is classified.
genus	The scientific name of the genus in which the taxon is classified.
specificEpithet	Scientific name.
country	The country where the species occur.
stateProvince	State of Brazil where species occur.
municipality	Municipality of Brazil where species occur.
decimalLatitude	The latitude component (N/S) of the coordinates of the municipality where the species occur, in decimal degrees.
decimalLongitude	The longitude component (E/W) of the coordinates of the municipality where the species occur, in decimal degrees.

### Data set 3.

#### Data set name

Monocots_Brazil_Gomes_da_Silva_Forzza_Lanna.tsv

#### Data format

TSV

#### Number of columns

8

#### Download URL

https://ckan.jbrj.gov.br/dataset/e1eb798c-601a-4d20-bf17-87dc037ed73e/resource/4326f085-dbbe-48ff-812d-aba565f64c8d/download/monocots_brazil_gomes_da_silva_forzza_lanna.tsv

#### Description

Data containing the geographic distribution of 3,854 species of monocots from 32 families.

**Data set 3.** 

Column label	Column description
family	The scientific name of the family in which the taxon is classified.
genus	The scientific name of the genus in which the taxon is classified.
specificEpithet	Scientific name.
country	The country where the species occur.
stateProvince	State of Brazil where species occur.
municipality	Municipality of Brazil where species occur.
decimalLatitude	The latitude component (N/S) of the coordinates of the municipality where the species occur, in decimal degrees.
decimalLongitude	The longitude component (E/W) of the coordinates of the municipality where the species occur, in decimal degrees.

## Additional information

Despite the digitisation efforts of numerous museums and herbaria, data gaps remain. We strongly encourage and recommend that distributional data be correctly georeferenced in collections in order to increase the quality of the spatial data used in future analyses.

Due to the immeasurable importance of primary occurrence data and the difficulties in georeferencing inaccurate geographical distribution data, we recommend that collectors strive to prioritise and record exact coordinates for their collections (see discussion in Colli‐Silva et al. 2020). In addition, the sharing of georeferenced data should become standard procedure, in line with sharing DNA sequences data in GenBank. As well as the georeferenced data in the present work being returned to the REFLORA database, we recommend that small and large datasets of georeferenced data should be returned to the collections database and published in a data paper. Unquestionably this "standard procedure” will improve the quality of primary data and provide greater accuracy in future biogeographical analyses, thus promoting the advancement of science.
